# Lymphoedema - Filariasis or soft tissue sarcoma? A case report

**DOI:** 10.51866/cr.877

**Published:** 2025-07-29

**Authors:** Tamring Sarinah, Mat Nawi Zanaridah, Nik Yusof Fuad Nik Farah, Joon Hi Tham, Mohamad Setia Awang Shahrunizam

**Affiliations:** 1 MD, Klinik Kesihatan Putatan, Jalan Pasir Putih, Putatan, Sabah, Malaysia. E-mail: sarinaht89@gmail.com; 2 MBBS, Dr. Fam. Med, Klinik Kesihatan Putatan, Jalan Pasir Putih, Putatan, Sabah, Malaysia.; 3 MBBS, MMED (Fam. Med), Klinik Kesihatan Putatan, Jalan Pasir Putih, Putatan, Sabah, Malaysia.; 4 MBBCH(Bao), MPath, Hospital Queen Elizabeth, Jalan Penampang, Kota Kinabalu, Sabah, Malaysia.; 5 MBBS, DrRad, Fakulti Perubatan dan Sains Kesihatan, Universiti Malaysia Sabah, Jalan UMS, Kota Kinabalu, Sabah, Malaysia.

**Keywords:** Lymphoedema, Filariasis, Soft tissue sarcoma

## Abstract

This is a case report of a middle-aged man who presented with chronic unilateral lower limb swelling and a mass on the same side. Several important differential diagnoses were considered. Lymphatic filariasis was included in the differential diagnoses due to a history of travel to a district endemic with filariasis in Sabah. A final diagnosis of soft tissue sarcoma was provisionally made based on the magnetic resonance imaging findings and confirmed by histopathological examination as pleomorphic dermal sarcoma. Both conditions are unusual but important causes in primary care due to their association with poor morbidity and risk of mortality. Patient management should involve a multidisciplinary team, including primary care, public health, radiology, pathology and orthopaedics.

## Introduction

Chronic unilateral lower limb swelling can be caused by various factors. Several differential diagnoses are considered prior to further workup and referral. The causes vary from chronic venous insufficiency to lymphoedema or malignancy. This is a case report of a patient who presented with unilateral lower limb swelling and a mass on the same side and was subsequently diagnosed with soft tissue sarcoma (STS).

STS is a rare malignant tumour with a high risk of mortality. A unique subtype of rare STS is pleomorphic dermal sarcoma (PDS), which is undifferentiated histologically from atypical fibrous xanthoma (AFX) but behaves more aggressively and has a higher risk of mortality. The management of this condition is demonstrated in this case report.

## Case presentation

A 47-year-old man came for routine follow-up of diabetes mellitus, history of ischaemic stroke with right-sided hemiparesis, hypertension and dyslipidaemia at a primary care clinic. He was able to ambulate at home using a quadripod walking stick but had used a wheelchair when visiting the clinic.

**Figure 1:**
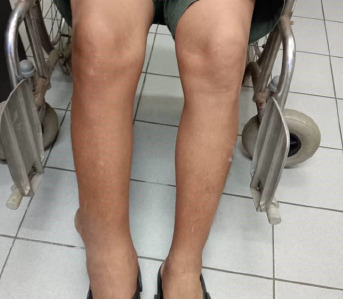
Anterior view of right lower limb swelling.

**Figure 2:**
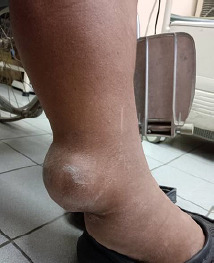
Posterolateral view of right lower limb swelling.

During this visit, the patient complained of right lower limb swelling associated with a mass over the posterior aspect of the right leg for the past 6 months (Figures 1 and 2). The localised swelling was painless, no history of trauma and it was slowly increasing in size. He denied any risk factor for deep vein thrombosis (DVT) and no constitutional symptoms were noted. His gait was not affected by the presence of the mass.

The patient’s hometown was from this district, but he had a history of travel to another district, which was a known endemic area of filariasis. He did not have any contact with any patient with filariasis or a history of screening or mass treatment for filariasis.

**Figure 3:**
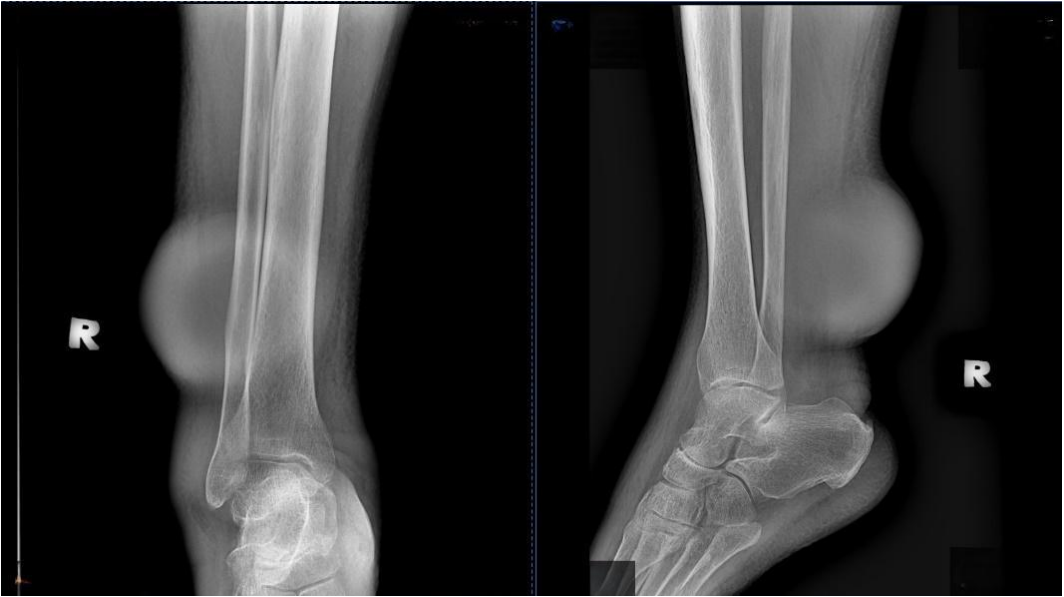
Radiographic view of the right lower limb (anteroposterior and lateral views).

Upon examination, there were diffuse pitting unilateral swelling of the right lower limb and another mass over the posterior aspect of the sight leg measuring 10x13 cm. The right leg circumference measured 43 cm, while the left leg circumference measured 41 cm. According; to Brunner’s classification, the patient had stage 1 oedema. There were no dilated veins over the affected limb. The mass was not pulsatile, and there was no bruit heard. The mass was located above the ankle on the posterolateral aspect of the right leg. It had a smooth surface and hard consistency, no skin changes and was non-tender but fixed to the structure beneath. The range of motion (ROM) of the right ankle joint was full. The slipping test and transillumination test revealed negative findings. No lymph nodes or intraabdominal mass was palpable. Radiography of the right tibia/fibula showed soft tissue shadow with no bone involvement (Figure 3).

The case was referred to the vector unit of the state health department. As the patient denied any risk factor of lymphatic filariasis, testing filariasia was not indicated at the moment.

The case was then referred to the radiology department and based on the input from the radiologist, magnetic resonance imaging (MRI) was indicated, with referral to the orthopaedic team as the primary team.

**Figure 4:**
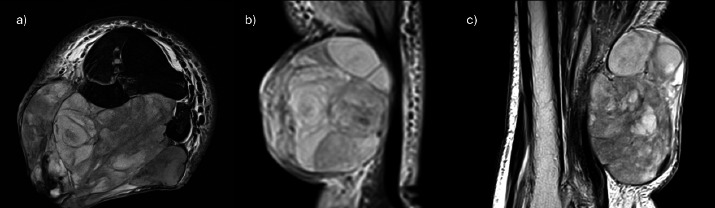

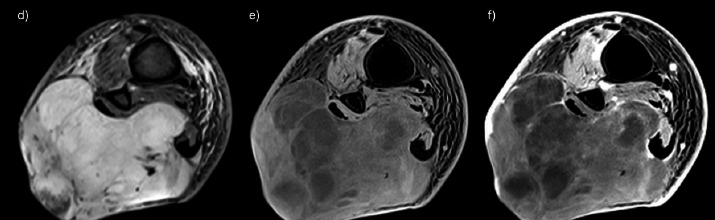
Magnetic resonance imaging (MRI) of the right leg. (a) Axial T2 weighted image. (b) (Coronal Short-Tau-Inversion-Recovery (STIR) image. (c) Sagittal T2 weighted image. (d) Axial Gradient Echo (GRE) image. (e)Axial fat-saturated T1 weighted image. (f) Axial fat-saturated T1 weighted image post-contrast

MRI of the right tibia/fibula revealed a large, lobulated and multiseptated heterogeneous mass at the subcutaneous layer occupying the right posterolaterd compartment of the right leg measuring approximately 8.1x10.2x9.6 cm. The moss wav heterogeneously hypointense on T1WI end T2WI sequences, with no significant cystic, Tat components or intralesional vascularity within. There was minimal blooming artifact, which may represent calcification or blood component. Heterogeneous enhancement was noted in the post-contrast study, in which the non-enhancing areas may represent necrotic component of the mass. There was no obvious intramuscular extension noted. The surrounding vessels demonstrated a loss of flow void likely due to mass effect causing slow flow (Figure 4).

An impression of STS needed to be ruled out via histopathological examination (HPE).

**Figure 5:**
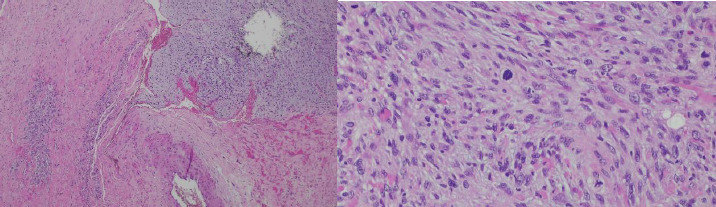
Pleomorphic spindle cell tumour (H&E stain, 4x and 40x objective magnification).

The orthopaedic team reviewed the case, including the magnetic resonance images and report. Subsequently the patient was scheduled for wedge biopsy on 27/11/24. HPE showed that the tumour infiltrated the entire dermis end was composed of markedly pleomorphic spindle to epithelioid cells, arranged loosely in sheets and fascicles. The mitotic count was 10 in 10 high power fields. Immunohistochemical stains were nonspecific. EMA showed single-cell positivity, and CD10 was diffusely positive, while AE1/AE3, SMA, S100 and SOX10 were negative. An impression of pleomorphic sarcoma (at least intermediate grade) was reported (Figure 5).

Computed tomography thorax-abdomen-pelvis (CT-TAP) showed right inguinal and para-iliac lymphadenopathy with no distant metastasis. The patient underwent right below knee amputation (BKA) on 11/12/24. HPE of the distal limb showed a clear margin of metastasis. Post-operatively, the patient was referred to the rehabilitation unit and orthopaedic oncology team for continuation of care.

During patient review at the primary care clinic at 1-month post-operative, the patient still required dressing over the BKA stump. He denied any complication such as phantom limb pain. Whooley’s questionnaire yielded negative findings. He denied feelings of low mood or anhedonia after BKA. At this moment, the patient was advised to do ROM exercises over the knee joint to maintain joint mobility with the target for prosthesis fitting in the near future. He denied any symptom to suggest distant metastasis such as dyspnoea or new mass recurrence.

## Discussion

STS is a rare type of malignancy with a poor 5-year survival. In general, a general practitioner may encounter one case of STS in their career lifetime.^1^ However, STS is considered the most common orthopaedic-related malignancy. According to Almas et al., the mean age of patients was 45.62±16.88 years,^2^ which correlates with this patient’s demographic background. A local study conducted at a single orthopaedic oncology centre reported that the median survival time was 10 years, while the 5-year overall survival rate was 58%, with the presence of distant and local metastases affecting this rate.^3^

A previous case series illustrated that delayed presentation affects the cancer prognosis. A separate case series from Tanzania reported three cases with initial symptoms presenting from 7 months to 5 years, causing the need to proceed to limb amputation.^4^ Thus, early suspicion from the first contact with primary care is important to expedite workup and referral.

According to the American Academy of Family Physicians, the following four signs are suggestive of malignancy: size greater than 5 cm, location on or below the fascia, fixation to surrounding structures and rapid growth or recurrence of a mass after prior excision.^5^ Patients with these features are indicated for expedited MRI with contrast, following radiologist recommendation. This patient presented two high-risk features, and MRI was performed after referral to the radiology team.

From a pathologist’s point of view, pleomorphic sarcoma represents a heterogeneous group of tumours with overlapping histologic features. These tumours have varying biologic potential, ranging from harmless to aggressively invasive and metastasising tumours.

Despite the abovementioned findings, definite tumour typing was not possible, as the specimen examined contained only the epidermis and dermis, without sufficient surrounding structures. It is rarely feasible to accurately type and grade a soft tissue tumour from an incisional or wedge biopsy.^6^

Based on the patient’s clinical history, examination, tumour histology and immunohistochemical stains, a diagnosis of PDS was favoured.

The potential differential diagnosis includes AFX, which is histologically identical to PDS. The contrast between AFX and PDS is stark: AFX is benign and treated with marginal excision, while PDS has the potential for metastatic spread and recurrence and requires wide excision and adjunct treatments.^7^

At present, treatment protocols for PDS are lacking based on few retrospective case series and case reports.^8^ A survey conducted in the United Kingdom found variations in opinions on management and pathways, including the involvement of a sarcoma multidisciplinary team, surgical excision margins and imaging at diagnosis and surveillance.^8^

Conversely, one important differential cause for lymphoedema in Sabah is filariasis. Sabah is among the states with an endemic area for filariasis besides Sarawak, Terengganu, Kelantan, Pahang, Selangor and Johor.

Filariasis is a tropical disease caused by filaria and transmitted by mosquitoes as vectors. Lymphatic filariasis is commonly caused by Wuchereria bancrofti sp. besides Brugia malayi sp. and Brugia timori sp. It is not a fatal disease but can cause disfigurement, potentially leading to depression, difficulty in securing employment and stigma.^9^

Filariasis is diagnosed based on the presence of microfilariae in blood smears or a positive result from the Brugia Rapid Test Kit. In 2000, the WHO introduced the Global Programme to Eliminate Lymphatic Filariasis. This programme consists of preventive chemotherapy in the form of mass drug administration and morbidity management to alleviate suffering among patients with lymphatic filariasis. Malaysia has yet to achieve elimination of filariasis as a public health threat.^10^ Thus, a high index of suspicion is necessary to identify and report suspected cases of filariasis.

## Conclusion

In patients presenting with chronic unilateral lower limb swelling, uncommon but important differential diagnoses need to be considered. Lymphatic filariasis is an important cause of unilateral lower limb swelling in Sabah, while STS is important malignancy due to its poor prognosis. Four clinical signs suggestive of malignancy warrant expedited imaging such as MRI prior to referral to the primary team.
